# Malaria treatment policy change in Uganda: what role did evidence play?

**DOI:** 10.1186/1475-2875-13-345

**Published:** 2014-09-02

**Authors:** Juliet Nabyonga-Orem, Freddie Ssengooba, Jean Macq, Bart Criel

**Affiliations:** WHO Regional Office for Africa, P.O Box 6, Brazzaville, Congo; Makerere University, School of Public Health, P.O. Box. 7072, Kampala, Uganda; Université Catholique de Louvain, Boite 3058, Clos Chapelle aux champs, 30, 1200 Bruxelles, Belgium; Institute of Tropical Medicine Antwerp-Belgium, Nationalestraat 155, 2000 Antwerp, Belgium

**Keywords:** Malaria treatment policy change, Evidence, Knowledge translation

## Abstract

**Background:**

Although increasing attention is being paid to knowledge translation (KT), research findings are not being utilized to the desired extent. The present study explores the role of evidence, barriers, and factors facilitating the uptake of evidence in the change in malaria treatment policy in Uganda, building on previous work in Uganda that led to the development of a middle range theory (MRT) outlining the main facilitatory factors for KT. Application of the MRT to a health policy case will contribute to refining it.

**Methods:**

Using a case study approach and mixed methods, perceptions of respondents on whether evidence was available, had been considered and barriers and facilitatory factors to the uptake of evidence were explored. In addition, the respondents’ rating of the degree of consistency between the policy decision and available evidence was assessed. Data collection methods included key informant interviews and document review. Qualitative data were analysed using content thematic analysis, whereas quantitative data were analysed using Excel spreadsheets. The two data sets were eventually triangulated.

**Results:**

Evidence was used to change the malaria treatment policy, though the consistency between evidence and policy decisions varied along the policy development cycle. The availability of high-quality and contextualized evidence, including effective dissemination, Ministry of Health institutional capacity to lead the KT process, intervention of the WHO and a regional professional network, the existence of partnerships for KT with mutual trust and availability of funding, tools, and inputs to implement evidence, were the most important facilitatory factors that enhanced the uptake of evidence. Among the barriers that had to be overcome were resistance from implementers, the health system capacity to implement evidence, and financial sustainability.

**Conclusion:**

The results agree with facilitatory factors identified in the earlier developed MRT, though additional factors emerged. These results refine the earlier MRT stating that high-quality and contextualized evidence will be taken up in policies, leading to evidence-informed policies when the MoH leads the KT process, partnerships are in place for KT, the WHO and regional professional bodies play a role, and funding, tools, and required inputs for implementing evidence are available.

**Electronic supplementary material:**

The online version of this article (doi:10.1186/1475-2875-13-345) contains supplementary material, which is available to authorized users.

## Background

Although commitment to knowledge translation (KT) has been an issue of interest to funders of research, researchers, and policymakers, there is a concern that research findings are not being utilized to the extent that they should [1–4]. Several studies have documented the barriers and facilitatory factors for the uptake of evidence in policy development and many lessons have been learned [5–9]. Delays in using evidence to change treatment protocols, which in some instances have been longer than seven years [7], have led to wasting resources due to the continued use of ineffective care, with suboptimal health outcomes. Among the documented reasons for such delays is the poor quality of available evidence, political processes lacking inclusive dialogue, donor influences, a lack of openness to using evidence by policy makers, lack of required inputs to implement the evidence, limited alternatives, and concerns regarding the duration over which the new drugs will remain efficacious [5, 6, 10]. Available models on improving the uptake of evidence in policy development only marginally address these factors, some of which specifically pertain to low-income countries (LIC) [5, 10–12]. Scholars have pointed out the specificity of KT processes, stating that they are influenced by the nature of the policy, context, and stakeholders involved [12, 13].

In the present study, evidence is broadly defined to include research study results (both published and unpublished), findings from monitoring and evaluation (M&E) studies and population-based surveys, Ministry of Health (MoH) reports, community complaints, and clinician observations [14, 15]. The term KT is defined as a dynamic and iterative process including the synthesis, dissemination, exchange, and ethically sound application of knowledge to improve health, strengthen the healthcare system, and provide more effective health services and products [16]. In the present study, the terms “uptake of evidence in policy” and “knowledge translation” are used interchangeably.

This study, which looks at the uptake of evidence in policy development, specifically in reference to changes in the malaria treatment policy and its implementation, is part of a larger study exploring ways to improve KT in Uganda. Previous work in Uganda led to the development of a middle range theory (MRT) outlining the main facilitating factors for translating evidence into policymaking [17]. MRTs are defined here as “theories that lie between the minor but necessary working hypotheses (…) and the all-inclusive systematic efforts to develop a unified theory that will explain all the observed uniformities of social behaviour” [18].

The MRT detailing facilitating factors to the uptake of evidence as identified by policy actors in Uganda states the following:

“*High-quality and contextualized evidence will be taken up in policies so as to lead to evidence-informed policies in instances where the MoH leads the KT process and there are partnerships for KT in place.*

Evidence must be of high quality, contextualized, providing economically feasible recommendations, and produced in a timely manner by credible researchers. Use of local researchers is helpful but there is need for separation of roles between researchers and policymakers.

KT requires strengthened MoH institutional capacity to lead the KT process. Institutionalized platforms for engagement between researchers and policymakers including civil society need to be in place, and mechanisms to coordinate evidence generation and synthesis need to be mainstreamed within the MoH. The capacity of policy makers in knowledge management needs to be strengthened and the policy making process need not be very bureaucratic.

Partnerships for KT need to be in place and all relevant stakeholders must be involved throughout the process to improve trust and build interest. Communities need to be involvement in evidence generation and KT as well.

*These contribute to higher ownership, adoption, and better application of evidence* [17] *”.*

The MRT was developed on the basis of a literature review and then validated with policy actors in Uganda. The facilitating factors were collected from respondents without a specific reference to a given research project and policy outcome; the extent to which they are valid in other settings needs to be tested in specific policy case studies. This study explores the place of evidence in the design and implementation of the change in malaria treatment policy in Uganda using a case study approach. Specifically, the study seeks to assess the extent to which the previously developed MRT explains the uptake of evidence in policy development from a policymaking perspective and explore the barriers and facilitatory factors to the uptake of evidence in the malaria treatment policy change. Eventually, the application of this MRT to concrete, selected health policy cases will contribute to refining and enriching the previously developed MRT.

### Background to the case study

The background to this case study was published by Nanyunja *et al.* [19]. The increasing resistance against chloroquine (CQ) in the late 1990s in several African countries, as reported by the East African Network on Monitoring Antimalarial Treatment (EANMAT), caused concern [20]. EANMAT was established as a platform to bring together malaria researchers and policy-makers from the Ministries of Health of the three East African countries: Kenya, Uganda, and Tanzania. In Uganda, the MoH set up several sentinel sites in 1997 with support from EANMAT and the World Health Organization (WHO) to monitor the efficacy of anti-malarials. The sentinel sites represented all geographic, epidemiological, and ecological strata of malaria in Uganda. Evidence from these sentinel sites showed that resistance to CQ exceeded the WHO-recommended threshold beyond which a policy change is recommended [21, 22]. Thus, several countries, including Uganda, embarked on changing their malaria treatment policies [7, 10, 19, 23, 24]. A review of this process in Uganda highlighted the importance of managing the policy change process, generating and using evidence for policy decisions, and the availability of adequate and predictable funding for effective policy roll-out [19]. The malaria treatment policy initially changed from CQ only to a combination of CQ/sulphadoxine/pyrimethamine (SP) in June 2000. Due to increasing resistance to CQ/SP, the treatment policy was changed again to artemisinin combination therapy (ACT), specifically artemether-lumefantrine (AL) (trade name Coartem®), as the first-line treatment for uncomplicated malaria, with artesunate-amodiaquine (AS/AQ) as an alternative [25]. The process occurred over a period of 25 months, from March 2004 to April 2006.

## Methods

### Study design

The case study approach was used based on the need to understand complex contextual issues [26]. The case is the malaria treatment policy change from CQ/SP to AL with AS/AQ as an alternative first-line treatment, which occurred in Uganda within a time span of 25 months between March 2004 and April 2006. Case study research has been shown to offer an opportunity for detailed contextual analysis of real life situations when the boundaries between the phenomenon under investigation and context are not clearly evident [26]. Furthermore, several researchers have used case studies to test theories in real life situations [27, 28]. The validity of the results was enhanced through the use of multiple data collection methods and member checking [29]. Prior to finalization, the preliminary results were reviewed by stakeholders who were central to the policy case: two from the WHO and two from the MoH. Recall bias was ameliorated by interviewing a wide range of knowledgeable stakeholders and by using multiple data sources [29]. The case study was performed between June 2012 and August 2013.

**Figure 1:**
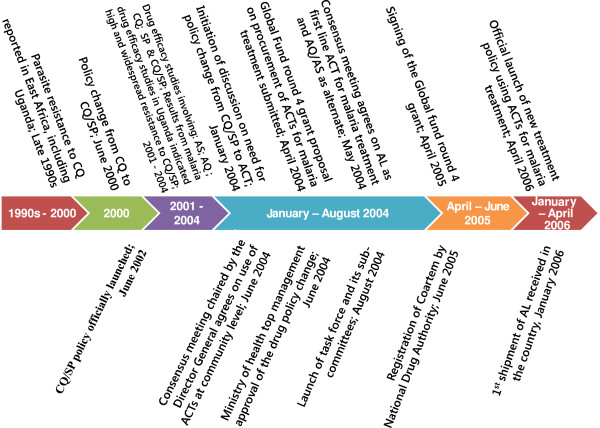
**Timeline of key events in changing the malaria treatment policy.**

In a quest to improve the comprehensiveness and validity of the findings, the present study employed both qualitative and quantitative methods (QUAL + quant) which are increasingly being applied to the investigation of complex issues in health systems research [30, 31]. A timeline of key events was developed based on a review of documents in consultation with two persons from the WHO and two persons from the MoH who held malaria-focused positions for over 10 years. This timeline guided the identification of key milestones, involved processes, the key documents to be reviewed, and the institutions involved, which subsequently informed the selection of respondents (Figure 1).

### Selection of respondents

The selection of respondents was guided by the study design. Using the timeline of key events, institutions were identified that were involved in the policy process. Key informants (KIs) were selected using purposive sampling, with the main criterion being their involvement in the research, design, or implementation of the malaria treatment policy change [32]. From each of the key institutions, the focal persons involved in the policy change process were selected and employed the snowballing technique to identify other key respondents until saturation. Some of the identified respondents had since moved on to other employment or retired and were categorized under the institutions they worked for at the time of the policy change. The identified focal researchers were selected for interviews if they had been involved in malaria research and provided evidence that was considered in the policy change process. Emphasis was placed on collecting their perceptions in line with the study questions, beyond what they may have published in scientific papers and research reports.

To obtain perceptions from across the spectrum of the healthcare delivery system, two districts with high malaria endemicity [33] were purposively selected based on proximity and the presence of a regional referral hospital (Jinja district) or general hospital (Mpigi). Within these districts, two hospitals and two lower level facilities (one public and one private not-for-profit in both districts) were purposively selected based on proximity and the desire to include different levels of the healthcare system. At the district level, the district health officer and a member of the district health team in charge of supervising health facilities within the district were purposively selected. Finally, the medical superintendent, or health centre employee in charge, and one clinical staff member responsible for the outpatient department at each health facility, were purposively selected as these employees interface with patients on a daily basis and are more likely to know the malaria burden, community health-seeking behaviours, and to have interfaced often with the supervising teams.

The selected respondents included donor representatives, public policy makers, civil society organizations (CSOs), researchers, the media, and representatives of the pharmaceutical sector. Managers of health services at the district level, health care providers from the public and private not-for-profit health facilities, senior officials from the national medical stores (NMS) in charge of medicine procurement and distribution, and managers from the national drug authority (NDA) in charge of medicine regulation were also interviewed. Details of the selected respondents are shown in Table 1.

**Table 1:** **Key informant respondents**

	Institution	Number of respondents	Average number of years in post
	Donors	3	8
Public sectors	National level MoH	10	11
	National medical stores (NMS)	1	3
	National drug authority (NDA)	1	6
	Service providers	4	7
	Managers at district level	2	9
	Researchers in universities	1	8
Private sectors	Civil society organizations	3	9
	Researchers from private research institutions	1	7
	Media	1	8
	Private pharmaceutical sector	1	5
	Service providers&	3	6
**Total number of respondents**		31	

&One of the selected districts did not have a private not-for-profit hospital.

### Selection of relevant documents

The timeline of key events guided the identification of relevant documents to be reviewed. A broad range of documents relevant to the case were included in order to ascertain the processes and stakeholders involved and assess the use of evidence.

### Qualitative research methods

The qualitative part of the study assessed whether evidence was available to guide policy decision-making, the nature of the evidence that was available, the extent to which evidence was disseminated and discussed in relevant forums, whether and why evidence had or had not been considered in policy development and implementation, and whether policy decisions were in line with the available evidence. The barriers and facilitatory factors to the uptake of evidence in the malaria treatment policy change and implementation process were explored.

### Data collection

Data collection methods included a review of documents and interviews with KIs. Interviews were conducted with KIs using an in-depth interview guide consisting of open-ended questions. The interview guide was developed by the first author and was reviewed and refined by the research team prior to pretesting it with volunteer colleagues in the WHO Uganda office (n = 2), technical officers in the MoH (n = 2), and one researcher from the Makerere University School of Public Health in Uganda. KIs were contacted and invited by email or telephone to participate in the study. All identified respondents accepted to participate and were interviewed. All interviews were conducted by the first author face-to-face in English (see Additional file 1).

Relevant documents were reviewed using a review guideline and included MoH position papers, concept papers, minutes of meetings, malaria policies and guidelines, research reports, malaria proposals to the Global Fund, reports of working groups, and supervision and research reports as identified over the timeline of key events (see Additional files 2 and 3).

### Data analysis

Interviews were recorded, transcribed verbatim, and entered into MS Word software for editing as the first step in the “formal” analysis. The interviews lasted 45 minutes on average. During the interviews, the first author made additional notes of initial findings and impressions, which were used to enrich the transcribed interviews. Next, the first author read all of the transcribed interviews to identify emerging issues, and then the first two authors analysed the interviews together to identify emerging issues. Deductive content thematic analysis [34] was used to organize the emerging issues under themes in line with the initial MRT. An example of how the deductive content thematic analysis was conducted is provided in Table 2. The research team then reviewed and interpreted the findings. Converging issues were reviewed again by the research team and, when interpretations differed, consensus was achieved by revisiting the raw data and discussions. Identified regularities were compared with the previously developed MRT to identify convergent and other emerging issues. Similarities and contrasts between respondent perceptions were reviewed by the research team and possible explanations for the contrasting views discussed. When necessary, quotations that best represent the emerging issues were edited slightly for flow while preserving the meaning of the text.

**Table 2:** **An example of content thematic analysis**

	Category 1 (manifest)	Category 2	Subtheme	Theme
Evidence used was mainly on the efficacy of the drugs being used (CQ/SP) and there was good quality evidence from different sites in the country showing that the efficacy of the drugs was declining.	Evidence used was mainly on the efficacy of the drugs	Efficacy studies of high quality from multiple sites showing consistent results	High-quality evidence of drug efficacy (CQ/SP)	**Characteristics of available evidence**
	Good evidence from different sites in the country			
Though few studies has been done in Uganda, there were studies in other countries in the region- Ghana, Zambia- showing high levels of efficacy for the ACTs in a similar environment. The evidence from the clinical studies was quite good- the studies were comparable and had been done with adequate sample sizes. The data were consistently showing increasing resistance.	High-quality evidence showed a high level of efficacy for the ACTs in other similar settings			
A lot of international evidence on efficacy was used, including sentinel surveillance sites set up by EAMAT, who are respected professionals.	Evidence from MoH-owned sentinel studies supported by respected researchers			
There was not overwhelming data in the country to compare the two (CQ/SP & ACTs); but real data was available showing that CQ was failing. Just a few studies compared ACTs, but the particular ACTs being used and that were recommended as the best for the circumstances had only been investigated by one study in Uganda.	Real data showing CQ was failing			
There was also the option of taking amodiaquine artesunate and artemether lumefantrine. A few studies investigated artesunate, but studies with amodiaquine showed that amodiaquine resistance was not yet at the mark where it cannot be used, especially if it is in combination with SP, but the resistance of amodiaquine was increasing rapidly. Then the issue was that, if combination therapy is used and amodiaquine loses efficacy in the next one to two years, the problem of mono therapy will return.	Efficacy data was available on the different options: amodiaquine artesunate and artemether lumefantrine	Locally available evidence on drug efficacy study discussed in a stakeholder forum	Locally available evidence of the efficacy of other potential drugs/ACTs was reviewed and discussed	
As a country, none of the partners involved in implementing malaria control activities disagreed with the results on CQ resistance that came out of the studies.	All partners agreed with research results		Consensus on research results	

### Quantitative research methods

Quantitative methods were used to capture the multiple perspectives of the involved stakeholders and enable the identification of regularities and patterns [35]. The quantitative part of the study measured the frequency with which evidence was cited in reviewed documents (including different types of evidence) and the respondents’ rating of the degree of consistency between the policy decision and available evidence. A policy development framework including the steps of agenda setting, policy formulation, selection of preferred options, and implementation [36] was used to organize the quantitative part of the case study.

### Data collection

The document review entailed quantifying the frequency with which evidence was cited, including the type of evidence (local *versus* international research; operational research, systematic review, basic research, M & E data). Using a semi-structured questionnaire, KIs were asked to rate the consistency between policy decisions and available evidence. The consistency between evidence and policy decisions were rated using scales developed by Hanney *et al.* [3], which rate different parameters on a scale of 1 to 4 (1, considerable level of agreement; 2, moderate level; 3, limited level; 4, no indication of consistency despite availability of evidence). In applying the scales, the factors taken into consideration included the degree to which the policy was consistent with evidence in terms of the definitions of the policy problem and objectives and the description of the strategies and actions, and how far the elements of the policy contradicted the available evidence.

### Data analysis

Quantitative data were analysed using Excel spreadsheets. Qualitative and quantitative data sets were eventually triangulated. In addition, findings from the document analysis and the analysis of KI interviews were integrated throughout the analysis. Informed consent was obtained from all respondents prior to the interviews. Study participants were informed about the purpose of the study and the scope of issues in the in-depth interview guide. Confidentiality was ensured in data management and only aggregate information without subject identifiers is reported. All data were secured in a safe location accessible only to the study team. Ethical approval was obtained from the Institutional Review Board of the Institute of Tropical Medicine, Antwerp (Belgium; IRB number IRB/AC/ac/197) and Uganda National Council for Science and Technology (number SS 2920).

## Results

### Use of evidence in changing the malaria treatment policy

The change in malaria treatment policy was reported to be very technical, and in which the role of evidence was very important. A civil society respondent stated more specifically that, “*This is mainly scientific because we are dealing with technical issues, hence no room for guessing****.****As civil society, although we reach out mainly to communities, we were at task to explain the need for a policy change using scientific explanations”.*

The evidence that was reported to have been available and considered in the policy process was categorized into nine areas (Table 3), namely local and international evidence on drug efficacy, guidance from the WHO, “cries” from the community, evidence on cost, implementation feasibility, routine monitoring data, local and international experiences, observational evidence, and evidence on behavioural change.

**Table 3:** **Type of evidence used to change the malaria treatment policy as reported by respondents**

		High-quality evidence on drug efficacy (CQ/SP and ACTs)	Guidance provided by WHO	Cries from the community	Evidence on cost	Implementation feasibility	Routine monitoring data	Local and international experiences	Observational evidence from clinicians	Evidence on behavioural change
	Donors	3	3	1	2	1	0	2	1	1
Public sector	Central level MoH	7	6		4	1	1			
	NMS									
	NDA	1	1						1	
	Service providers	1	2	2		1	1			
	Managers at district level	2	1				1			
	Researchers	1	1							
Private sector	CSO	1		1						
	Pharmaceutical company	1								
	Media	1		1				1		
	Service providers	1				2				
	Researchers	1	1	1		1			1	
	**Total**	**20**	**15**	**6**	**6**	**5**	**3**	**3**	**3**	**1**

Evidence on the efficacy of CQ/SP and ACT was reviewed, discussed, and guided decision-making, as highlighted by a donor respondent: “*We used evidence to change the malaria treatment policy. We mainly used evidence on the efficacy of the drugs that we were using (CQ/SP). There was good evidence from different sites in the country showing that the efficacy of the drugs was really declining*”. This evidence was deemed to be of high quality because multiple study sites were using a WHO-approved protocol. Technical support was provided by EANMAT and the WHO when setting up the sites and for the development of research protocols, data analysis, and interpretation. Results from the different sites were consistent, and the results were consistent with those of other countries with similar malaria endemicity:

“*The methodology to undertake studies on the efficacy of medicines was thorough, so when we presented the evidence, there were no loopholes. We made sure that results were not from only one study area. Otherwise, with the challenges we had with this malaria treatment policy shift, if there were loopholes in the data we would have been shot down, especially at the point when people started thinking that maybe some drug companies were the ones pushing it on Uganda and that the country was not going to afford the new policy*”. (Researcher respondent)

The WHO provided evidence from global and regional levels, as well as standards with regards to drug resistance cut-off levels at which a country should embark on changing their malaria treatment policy. This was important in guiding decision-making, as emphasized in the following quotes:

“*WHO is seen as the authority on clinical matters; when the WHO takes a stance and says that this drug is better for patients than the old drug, the country will often take that recommendation very seriously*”. (Donor respondent)

“*Uganda is a member of the WHO - so we tend to take advice and guidance from the WHO. Whenever WHO updates guidelines or sends out new information, we take that advice. One of the reasons why a country, like for example Uganda, should change the policy is because they are being guided by an international body like the WHO, which is our technical arm in policy around malaria and other diseases*”. (MoH respondent)

Cries from the community further created impetus to take action, as one donor respondent stated, “*There was a public outcry, and the public outcry comes in many forms, either through newspaper articles or, particularly if you are the health worker, people will tell you ‘doctor you gave me this drug for a fever last week and it is not getting better!’ That is how they communicate to you their concerns, and that is strong enough. So to me, I think that is strong enough evidence and we used it”*.

At the implementation stage, community complaints, which referred to perceptions of the effectiveness of Coartem®/AL, were used to address operational challenges:

“*When we came in with Coartem, three months later we started getting complaints from the community that the new drug was not as effective as CQ; when you take it you still feel weak and the temperature does not go down. The problem was not that the new drug was not working, but it did not have the property of reducing temperature. So we had to go on radio to make the announcements nationally saying ‘when you are taking Coartem*®*, take an antipyretic like Aspirin or Panadol together with Coartem,’ and it worked*”. (MoH respondent)

Routine M & E and evidence from supervision reports were also used at the implementation stage. However, reservations were expressed by service providers regarding the quality of routine data stating that “*we submit our monthly reports to the District Health office, and these reports are used in assessing delivery of services, including malaria, but I have never seen any change. No feedback to improve the quality of data*”.

### Facilitatory factors for the uptake of evidence

Respondents identified factors that facilitated the uptake of evidence, and these were categorized into five themes: 1) characteristics of the available evidence, 2) MoH institutional capacity to lead the KT process, 3) partnerships for KT, 4) availability of tools and inputs to implement evidence, and 5) intervention of the WHO. These themes are summarized in Table 4 and the details of each theme are provided in Additional file 4.

**Table 4:** **Factors that facilitated the uptake of evidence as reported by respondents**

	Donors	MoH	NMS	NDA	Service providers	CSO	Pharmaceutical company	Media	Researchers	Total
Characteristics of available evidence	10	20	2		14	4	4		3	57
MoH institutional capacity to lead the KT process	1	8		2	2	3			4	20
Partnerships for KT	3	7		1	3	2	1		1	18
Availability of tools and inputs to implement evidence	2	5			9	1				17
WHO intervention	3	4				1			1	9

### Characteristics of the available evidence

The characteristics of available evidence encompassed several dimensions, including the availability of high-quality local evidence, the availability of competent in-country researchers, consistent results from multiple studies performed by different researchers, evidence generated by credible international researchers/regional network, and consensus on research results.

High-quality local evidence on the efficacy of CQ/SP was available from sentinel sites set up in Uganda by the MoH with support from EANMAT and the WHO. A private pharmaceutical representative noted that “*the study design also influenced the uptake of evidence. For example, they used the WHO tools; people will easily accept such evidence other than coming up with individually designed tools. The WHO tools are already tested methods*”.

Some research studies were performed by competent in-country researchers who were deemed credible, and this helped the acceptance of results, as highlighted in the following quote from a donor respondent:

“*Uganda is fortunate that it has a lot of leading thinkers in malaria who know a lot about malaria. They were part of the process and were leading in all the studies. The availability of local data made it easier for running this case through the different levels of policy formulation, which would be different I suppose if you were in a country where there are no systems for research*”.

International researchers who had generated evidence from outside the country were also credible, as the MoH respondent stated, “*The regional network EARMAT is highly respected as a body of senior malaria experts, their support to sentinel sites in the regions gave a lot of credibility to the data, and they have been very supportive in terms of helping us to understand what is in the background*”.

Consistency in the research results of different study sites in the country and studies from other East African countries was a key factor, as stated by a donor respondent, “*Evidence sometimes is not easily accepted, but there was consistency in results from different sites. If you have several studies from different settings undertaken by different researchers showing the same results, it is easily accepted”.*

Evidence was disseminated through the media, as a MoH respondent stated, “*I remember we had a report coming out every Monday in the newspapers publicising the malaria burden*”. However, some felt that the extent to which evidence influenced decision-making could not be ascertained. A journalist remarked, “*We certainly published a number of stories on drug resistance in the newspapers although I am not sure to what extent those publications influenced decisions*”.

Similarly, the review of documents pointed to the availability of contextualized evidence on the efficacy of anti-malarials from studies performed in the country. Other sources of high-quality evidence included Demographic Health Survey (DHS) data supported by Macro International, the census and national household survey employing internationally agreed upon methodologies, and malaria economic studies, which were carried out in several countries following the WHO protocol.

There was separation of roles, the researchers conducted the studies and policy makers played a leadership role, receiving and discussing results. Research findings were discussed in several partnership forums. Evidence was discussed in the Malaria Case Management Technical Working Group (MCMWG)^a^, the Interagency Coordination Committee for Malaria^b^, and the national stakeholder forum. A research committee was put in place and included Malaria programme staff, researchers, and representatives of technical partners (WHO, Malaria Consortium, UNICEF). Research priorities were identified in a meeting that brought together all relevant stakeholders.

### MoH institutional capacity to lead the KT process

The MoH institutional capacity to lead the KT process encompassed several dimensions, including leadership role, the willingness of MoH to use evidence, MoH involvement in research studies, and a culture of the MoH using evidence to change treatment policies.

The strong leadership of the MoH and a culture of using evidence in policy development, facilitated partly by previous experiences, were echoed by several respondents. A researcher remarked, “*I recall that around the time that we transitioned from CQ/SP to ACT, the NMCP was in the hands of two people; both of them believed in evidence*”.

There was close collaboration between researchers and MoH policy makers, as reported by a researcher:

“*Our research programme has been working closely with the MoH and that helps; although were not always on the same page we had a good relationship. The sentinel sites were established by the malaria control programme a long time ago and we are using them. We have continued that relationship and, when we do studies, we often discuss with them what we are going to do and get a go ahead from the ministry. We have also had funding coming through the Ministry*”.

However, one donor respondent expressed reservations about the quality of MoH participation:

“*We have people in the ministry that are not well versed with the evidence they are using to decide whether this policy makes sense or not, and therefore you have people that think they know the evidence but really they do not*”.

The reviewed documents further confirmed that an institutionalized and systematic data collection system on drug efficacy was in place through the MoH-established sentinel sites (established in 1997). In addition, the Health Management Information System (HMIS), which collects data on service utilization and malaria cases, was also in place and managed by the MoH. These sources provided data that were referenced in the majority of the reviewed documents.

Furthermore, the MoH commissioned several studies, including a review of all available data, and closely followed up with researchers through the implementation of the research studies. Broad institutionalized platforms were in place for engaging all stakeholders. The MoH took leadership of the knowledge synthesis and application process through participation in, and chairing, the working group charged with synthesising all available evidence and making recommendations to the steering committee, which consisted of the decision-makers.

### Partnerships for KT

Partnerships for KT encompassed: the availability of platforms and structures within the MoH to discuss evidence, the interest of stakeholders to see that evidence is adopted into policy, and civil society involvement.

The availability of structures within the MoH to enable systematic dialogue was highlighted as a factor that improved the uptake of evidence:

“*At that time we had a very good team in the MoH and the opportunity to discuss things. There were systems and we had regular meetings and annual performance reviews. We had quarterly performance evaluations, which were very important and everyone had to be there right from the minister, so the climate for evidence-based decision-making, whether it was accidental or not, was there in the vision of the leadership*”. (MoH respondent)

Platforms to enable inclusive participation were also in place for evidence to be discussed, which facilitated consensus building, as highlighted in the following quotes:

“*The way the policy process worked is that the malaria programme in the ministry called together all its technical stakeholders - all its partners - everyone, government, academia, NGOs, etc. Everyone sat in one room and debated what they thought the best policy option should be. I thought this was an excellent process*”. (Donor respondent)

“*As a malaria programme, we have what we call a Malaria Case Management Technical Working Group, which periodically meets to review how our medicines are working in the country; so that is another arm which facilitated the uptake of evidence because we are able to detect a need for change*”. (MoH respondent)

**Figure 2:**
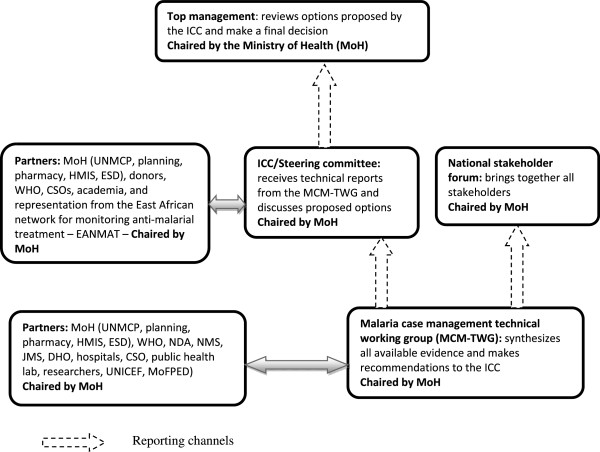
**Partnerships for decision-making.**

**Figure 3:**
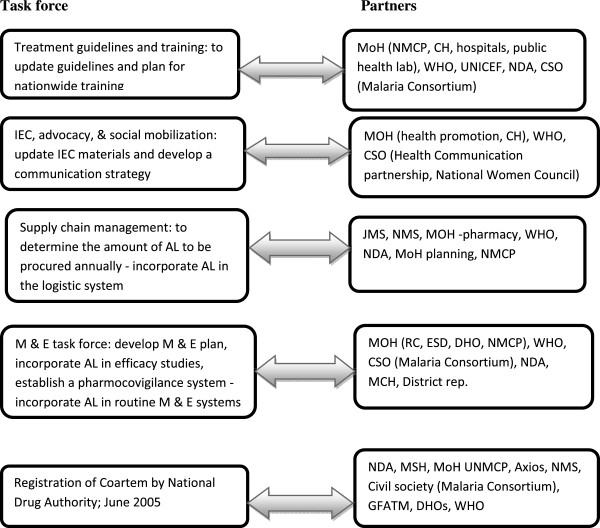
**Partnerships for developing operational tools and implementation details.**

The document review showed that partnerships were put in place at several stages of knowledge generation, synthesis, and application, bringing together relevant stakeholders. Figure 2 shows partnerships that were put in place for decision-making, whereas Figure 3 shows partnerships that were put in place for developing operation tools and implementing details.

The MCMWG ^c^ was put in place to synthesize available data and make evidence-based recommendations to the steering committee and MoH. The MCMWG concluded that, “After careful consideration of the evidence for CQ/SP treatment failure, the meeting agreed that there was need to change the anti-malarial drug policy (AMDP). In line with June 2000 recommendations to adopt a long-term policy and WHO recommendations, ACT was considered the most viable option to change to.” A policy decision that conformed to the available evidence.

Ahead of the national consultative meeting, evidence on drug resistance against the first-line treatment for malaria, as well as its interpretation and implications, was also presented to influential stakeholders in a form that was easy to understand. A national consultative stakeholder forum was then held, bringing together researchers, policy makers, clinicians, donors, CSOs, and district level health managers, and evidence synthesized by the MCMWG guided the dialogue. The national stakeholder forum identified information gaps, enabling all partners to participate in setting the research agenda. The meeting identified several studies covering several aspects to provide comprehensive evidence on cost-effectiveness, acceptability, and the feasibility of implementation. These studies were then commissioned by the MoH.

A series of meetings were held ensuring sustained dialogue throughout the policy change process. The steering committee provided regular briefings to members of MoH top management to apprise them of the situation, including presentations of synthesized evidence.

After a decision was made to change the first-line treatment for uncomplicated malaria to AL, the MoH commissioned task forces that brought together relevant stakeholders to work out the implementation process and mainstream implementation of the new policy in the routine system (Figure 3). In a meeting convened by the National Drug Authority (NDA), a decision was made to ban the importation of CQ and other monotherapies based on available evidence. The meeting agreed to strengthen linkages between the NDA, local researchers, and sentinel sites, to access data in order to prove and monitor drug efficacy, and to enable policy changes from time to time.

All stakeholders were represented in the different working groups and worked together to review the evidence, make policy decisions, and produce operational tools. All of the task forces were chaired by senior MoH officials.

### Availability of tools and inputs

The provision of guidelines, medicines, and training for health workers on the new policy are factors that favoured the implementation of evidence.

A MoH official stated that “*some health workers were totally green about the new anti-malarials; so we developed training materials and we trained health workers throughout the country.*” A service provider stated that “*the MoH provided us with guidelines which were very useful when it came to actual implementation of the policy. The civil society helped us to print more copies of these guidelines and provide them to health facilities*”.

Affordability issues were also raised, despite the availability of evidence. Some respondents reported that the decision to move to ACT was influenced by the availability of funding from the Global Fund (GF), as highlighted in the following quotes:

“*Around that time, there were was an opening for the Global Fund, and the possibility of applying for the Global Fund round 2 to support the policy change was raised. That was the only way we could realize the change to the new drug, and when we applied for funding and got it, that drove the decision*”. (MoH respondent)

“*There was the Global Fund at that point, otherwise it would have taken us a long time to change the treatment policy if we were to foot it alone. The Global Fund came on board with rounds where we had to make proposals to get funding for the new treatment, and when they gave us funds, we took up the policy*”. (MoH respondent)

The document review showed that the MoH put in place a mechanism to enable the implementation of evidence; the four task forces shown in Figure 3 were charged with the responsibility of developing operational instruments to implement the new policy, including mainstreaming the implementation of monitoring into routine systems. The operational tools also incorporated the evidence.

### Barriers to the uptake of evidence

Respondents mentioned resistance from implementers as one of the barriers that had to be overcome. One MoH official remarked that “*there is a general tendency of human beings resisting change just by nature, because they have learned the old ways of giving medicine and nobody is willing to adjust. So there was a problem with some of our health workers’ attitude”.*

Another reported barrier was the influence of drug companies, as stated by a service provider, “*You know as part of market dynamics- you will find that once a product has been in the country for some time, there is a system which promotes its sale; so when a change is proposed, there is resistance because someone has been promoting a product which the company has been selling for some time. That is one arm that can bar you from adopting good evidence*”. Health system considerations and sustainability of the new policy were among the additional challenges. Concerns also existed about whether the supply chain system would ensure the availability of ACT and whether the country could sustain the policy from its own resources.

### Quantitative results

All 18 of the reviewed documents referenced evidence; the documents cited evidence 57 times. Each type of evidence was counted only once in each reviewed document. The evidence cited most was mainly locally generated evidence, as opposed to international, efficacy studies, M & E, and WHO guidance (Table 5).

**Table 5:** **Nature and frequency of evidence cited**

Nature of evidence	Local evidence&	International
Efficacy studies^d^	20	10
Monitoring and evaluation^e^	12	
Guidance from WHO^f^		11
Surveys (NHS, DHS, census)^g^	7	
Operational research^h^	5	
Social science^i^	5	
Epidemiology	4	
Clinical observation^j^	3	
Entomology	1	
**Total**	**57**	**21**

&Refers to evidence from Uganda.

^d^Data on the efficacy of used antimalarials (CQ, SP, amodiaquine). Results from the Tanzania study and three sentinel sites in Uganda. The Uganda sentinel sites were put in place and supported by the MoH.

^e^Mainly the Health Management Information System showing malaria burden.

^f^Recommendation on when to consider changing the first line treatment; resistance cut-off levels. ACTs are the most effective medicines available to treat uncomplicated malaria.

^g^National household survey data, Uganda poverty participatory assessment surveys, DHS, census. Citing population-based data on self-reported malaria cases, use of ITNs, health-seeking behaviour, and access to malaria treatment.

^h^Evidence of the areas requiring strengthening in the logistic system, health system weaknesses affecting delivery of malaria interventions, and implementation experiences on home-based management of fever.

^i^Evidence of economic burden of malaria, evidence of other malaria control strategies such as behavioural change issues, and acceptability of the different anti-malarials.

^j^Clinicians’ observations in Uganda national referral hospitals.

Respondents rated the degree of consistency between the available evidence and decisions at the different stages of the policy cycle, namely agenda setting, policy formulation, selection of preferred option, and policy implementation (Table 6). This rating of the consistence between evidence and decisions taken was intended to assess whether evidence played a bigger role at a particular stage of policy development than at other stages.

**Table 6:** **Respondents’ rating of the consistency between available evidence and policy decisions**

			Public sector			Private sector			
		Donors (n = 3)	MoH (n = 8)	NDA (n = 1)	Service providers (n = 12)	Pharmacist (n = 1)	CSOs (n = 2)	Researchers (n = 2)	Total
**Agenda setting**	Strong (1)	3	7	1		1	1	2	**15**
	Moderate (2)		1						**1**
	Weak (3)								
	No influence (4)								
**Analytical stage**									
	Strong (1)	2	3	1				1	**7**
	Moderate (2)	1	4			1	1		**7**
	Weak (3)							1	**1**
	No influence (4)		1						**1**
**Decision-making**	Strong (1)	2	2					1	**5**
	Moderate (2)	1	4	1			1		**7**
	Weak (3)		1			1		1	**3**
	No influence (4)		1						**1**
**Implementation**									
	Strong (1)	2	1						**3**
	Moderate (2)			1	5			1	**7**
	Weak (3)	1	6		5	1	1	1	**15**
	No influence (4)		1		1				**2**

The consistency between evidence and decisions was strongest at the agenda setting stage. Consistency was moderate in the analytical and decision-making stage and weak at the implementation stage.

## Discussion

Evidence played a key role in changing the malaria treatment policy; however, the level of consistency between available evidence and decisions varied along the policy development cycle. The characteristics of the available evidence, strengthened MoH capacity to lead the KT process, existence of partnerships for KT, and availability of tools to implement evidence were the most important facilitatory factors. Among the barriers to be overcome was resistance from implementers, the health system capacity to implement the new policy, and financial sustainability.

The characteristics of the available evidence, as reported in this study, have been shown to enhance ownership and the application of evidence [37, 38]. In addition, the separation of roles between researchers and policy makers, which is an identified factor that can safeguard scientific rigour and the objectivity of research, was respected [12, 17]. This partly explains the fact that, evidence guided decision-making. Outcomes have been different in cases where evidence was deemed to be of poor quality, contradictory, and inconclusive [39]. Mubyazi *et al.* reported delays in changing the malaria treatment policy because policy makers casted doubt on the available evidence [7]. Ssengooba *et al.* reported similar findings for the uptake of evidence on medical male circumcision as a preventive measure for HIV; the policy was delayed due to the president of the country discrediting research results [9].

The credibility of researchers, receiving support from the WHO, and utilising regional approaches as opposed to focusing solely on country efforts are also crucial facilitatory factors [40] realized in this study. Similarly, Woelk *et al.* documented the intervention of international researchers and involvement of local researchers in regional and international research networks as a facilitatory factor to the uptake of evidence in the magnesium sulphate trial for the treatment of pre-eclampsia [41]. These networks expose local researchers and clinicians to a culture of evidence-based decision-making.

Several types of evidence were employed, but the emphasis was on the efficacy of medicines. Although the use of evidence on the efficacy of medicines has been a common practice in most countries when changing malaria treatment policies, evidence is needed from several aspects for policy decision-making [10, 42]. In some instances, cost considerations and the feasibility of implementation have driven decision-making despite the availability of high-quality efficacy data [7]. In addition, community acceptability has affected the uptake of evidence in some cases. Malik *et al*. documented a community preference for drugs that they knew how to use and were cheap and affordable [24]. In the case of Uganda, the use of different ACT packs for different age groups was an identified challenge that affected the implementation of evidence [19]. This finding highlights the need for comprehensive evidence to guide decision-making and, in this study, evidence was available on health seeking behaviour, access to anti-malarials, the capacity of the supply chain system, community acceptability of anti-malarial interventions, the affordability of the new policy by the government, and the capacity of the health system to implement the changes.

The tailored dissemination to influential stakeholders, which occurred at the beginning of the policy process, could have influenced decision-making, as evidenced by the strong rating of the influence of evidence on decisions made at the agenda setting stage. Effective dissemination of evidence has been documented as a facilitatory factor to the uptake of evidence [43, 44]. However, the reduced consistency noted when moving from the analytical stage to implementation may not solely be the result of the dissemination modalities used; it may imply that other factors played a more central role than evidence. These are stages where issues such as affordability, health system issues, political will, and donor interest have influenced debates [45].

KT has been realized in instances where the MoH has the capacity to take a leadership role in coordinating the generation, synthesis, and application of evidence [46, 47], which was the case in this study. In their study of six countries in Southern Africa, Varkevisser *et al.* documented the successful uptake of evidence into policy where ministries of health led the process of evidence synthesis and dissemination [46]. In contrast, Lavis *et al.* documented successful KT when structures outside the ministries of health led the evidence synthesis and dissemination process, but these were in areas of clinical care and technology assessment [48]. In this case study, a two pronged approach was employed with mainstream structures, which have been shown to be beneficial given the opportunity to engage with stakeholders more effectively [47], and regional professional bodies, which have been shown to be very effective, especially for getting evidence into clinical practice [14, 48]. Some scholars have argued that mainstream structures are more relevant for getting evidence into public health policies [47], but these may also play a role in getting evidence into clinical practice, more so in low income countries where clinical decisions have far reaching health system implications. The presence of institutionalized platforms for KT, as existed in this case study, have been shown to be beneficial [3]. Ssengooba *et al.* also documented successful KT in Uganda where the uptake of evidence on the prevention of mother to child transmission was facilitated by the presence of KT platforms [9]. A long history of partnerships, as seen in this case study, helped build trust over the years and created a platform from which evidence could be discussed and decisions made. The involvement of relevant stakeholders in partnerships throughout the process, from knowledge generation to applications, was shown to enhance the uptake of evidence in policy development [17, 24]. Colon *et al.* documented a case in which the application of evidence was frustrated by weak partnerships engulfed in suspicions and protection of personal interests [49]. Although partnerships are beneficial, the realization of positive results in not always obvious, and among documented challenges is the varied capacity of stakeholders [40], effective leadership to encourage open dialogue and ensure respect [50], and time constraints on the part of decision-makers to allow the mobilization and participation of stakeholders. In this case study, these issues could have been ameliorated two ways. First, partnerships in policy making regarding malaria issues was a long-standing tradition. Second, partnerships in health policy development in general were already ingrained in the vision of the leadership, with participation at the highest level of the MoH.

Communities have been shown to be key stakeholders and, in this study, “community cries” created an impetus for policy change. Although effective community engagement remains a challenge in several low-income settings due to a lack of structures and resources [51], they are stakeholders in KT whose role could be maximized. The need for targeted dissemination to communities has been raised following the argument that, if they are armed with the right information, they can demand that certain policies be put in place [1, 52]. In this case study, the media played a role in disseminating evidence through newspapers, and this could have facilitated community involvement.

In this study, the provision of guidelines and medicines, training health workers on the new policy, and mainstreaming the implementation in routine processes favoured the implementation of evidence. Scholars have documented failed KT in several low income settings due to a lack of medicines, drug licensing, inadequate human resources, and lack of training [6, 7, 51, 53]. Kangwana *et al.* also documented challenges to implementing the revised malaria treatment policy due to a lack of ACTs [54], whereas Bergstrom *et al*. documented the failure of health workers to implement new knowledge due to a lack of required medicines and equipment [55]. Instances exist in which the affordability was the major factor guiding decision-making despite the availability of efficacy data, such as the case of changing the anti-malarial treatment policy in Tanzania [7]. This was also a key consideration in this case study as highlighted that … *there was the Global Fund at that point otherwise it would have taken us a long time to change the treatment policy*. Donor funding requirements have also influenced decision-making in other settings, such as the development of HIV care guidelines in Ghana [45].

The present results agree with the facilitatory factors identified in the earlier developed MRT on KT in Uganda, although additional factors and themes also emerged. The MRT was refined as follows:

*High-quality and contextualized evidence will be taken up in policies so as to lead to evidence-informed policies in instances where the MoH leads the KT process, the WHO and regional professional bodies play a role, partnerships for KT, and tools and required inputs are available to implement the evidence.*

Evidence must be of high quality, contextualized, provide economically feasible recommendations, and produced in a timely manner by credible researchers. Use of local researchers is helpful but there is a need for the separation of roles between researchers and policymakers. Effective dissemination of evidence to communities and tailored dissemination to influential stakeholders is also needed.

KT requires strengthened MoH institutional capacity to lead the KT process. Institutionalized platforms for engagement between researchers and policymakers, including civil society, need to be in place. Mechanisms to coordinate evidence generation and synthesis need to be mainstreamed within the MoH, although regional professional bodies also play a role and the WHO’s intervention is helpful. The capacity of policy makers in knowledge management needs to be strengthened and the policy making process must not be very bureaucratic.

Partnerships for KT need to be in place, and all relevant stakeholders must be involved throughout the process to improve trust and build interest. Building a culture of partnerships in health development over time enhances trust. Communities need to be involved in evidence generation and translation.

The availability of funding, tools, and inputs to implement evidence is crucial. Funding must be available, operational tools must be provided, and implementers must receive required training to implement the evidence.

*These contribute to higher ownership, adoption, and better application of evidence.*

### Strengths and weakness

The strengths of this study are the use of multiple data sources and interviews with a wide range of respondents, which generated a rich data set from which to assess the use of evidence. Among the weaknesses of this study is potential recall bias; interviews were conducted long after the policy development process occurred. However, the recall bias was ameliorated by the use of multiple sources of data. Rating has been attempted for the degree of consistency between available evidence and the decisions made at the different stages of policy development, but in reality the stages are not that distinct. The policy process is iterative.

## Conclusion

Different types of evidence were used in changing the malaria treatment policy in Uganda, though the level of consistency between evidence and policy decisions varied along the policy development cycle. Respondents perceived the availability of high-quality and contextualized evidence, including targeted dissemination of evidence to communities and influential stakeholders; MoH institutional capacity to lead the KT process; the intervention of the WHO and a regional professional network in evidence generation and policy development; existence of partnerships for KT with mutual trust; and the availability of funding, tools, and inputs to implement evidence as the most important facilitatory factors that enhanced the uptake of evidence in the malaria treatment policy change. Context is very important in KT, and the refined MRT may not hold in all contexts, but it can serve as a starting point for other countries planning to embark on changing their malaria treatment policies and seeking to maximize the use of evidence.

## Endnotes

^a^This was charged with spearheading all technical aspects of the change in the malaria treatment policy.

^b^This committee provided oversight over the work of the Malaria Case Management Technical Working Group.

^c^Technical committee - Malaria Case Management Technical Working Group (MCMWG) put in place to synthesize available data (technical committee) and make evidence-based recommendations to the steering committee and MoH. Malaria Case Management Group comprised the MoH (UNMCP, planning, pharmacy, HMIS, ESD), WHO, NDA, NMS, JMS, DHO, hospitals, CSO, public health lab, researchers, UNICEF, and MoF.

## Electronic supplementary material

Additional file 1: **In-depth interview guide for policy makers and researchers.** Open-ended question to elicit responses from respondents. (DOCX 15 KB)

Additional file 2: **Guideline for document review.** Guide used in reviewing documents. (DOCX 12 KB)

Additional file 3: **Documents reviewed.** Details of milestones and list of documents reviewed. (DOCX 24 KB)

Additional file 4: **Factors that facilitated the uptake of evidence in the malaria treatment policy change.** Details of facilitatory factors under the different themes. (DOCX 20 KB)

## Abbreviations

ACT: Artemisinin combination therapy
AL: Artemether/lumefantrine
AQ: Amodiaquine
CSOs: Civil society organizations
CQ: Chloroquine
EANMAT: East African Network on Monitoring Antimalarial Treatment
KT: Knowledge translation
MoH: Ministry of Health
MRT: Middle range theory
NDA: National Drug Authority
NMS: National Medical Store
SP: Sulphadoxine/pyrimethamine
WHO: World Health Organization.
