# Supplementary material for: Malaria treatment policy change in Uganda: what role did evidence play?
Source: Malar J. 2014 Sep 2;13:345. doi: 10.1186/1475-2875-13-345 (PMC4164770; doi:10.1186/1475-2875-13-345)
Supplement: Supplementary file 1 — Additional file 1: In-depth interview guide for policy makers and researchers. Open-ended question to elicit responses from respondents. (DOCX 15 KB) [file 12936_2014_3385_MOESM1_ESM.docx]

**In-depth interview guide for policy makers and researchers:**

The health sector in Uganda is underfunded and we need to use available funding optimally. This can only be achieved if we base our policy development and resource allocation decisions on available evidence and ensure that we put money where returns and maximized.

I would like to get your views on whether evidence has/has not been used and reasons why, level of importance, barriers and facilitating factors to uptake of evidence at the different stages of policy development. ***My focus is on malaria drug policy change and implementation.***

I have defined research broadly to include, research studies (both published and unpublished) and monitoring and evaluation studies, undertaken within the country by research institutions, universities, donors, civil society and government statistical units and; Ministry of Health reports.

You have been selected as one of the respondents because of your involvement in policy development/researcher in the areas of interest (malaria drug policy change and implementation).

May I use the recorder to record your responses?

Name of the interviewer: ________________________________________________

Date and time of the interview: ____________________________________________

*Details of the respondent:*

Name of respondent: _________________________________________________________

Title of respondent/Designation:__________________________________________

Stakeholder group/subsector respondent belongs to: ________________________________

Could you please briefly describe to me your current role and work responsibilities in this organization/institution *(probe for qualifications, research/policy involvement, and work experience*

Duration in that position: ________years; ___________months;______________

1. What do you consider as evidence suitable for informing policy development and implementation? (*probe for type of evidence, local vs international evidence, routine monitoring and evaluation, operational research, systematic reviews, social science, biomedical research*)
2. Has evidence been used in policy development and implementation with respect to malaria drug policy change and implementation? (*probe for use of evidence at the different stages; agenda setting; policy formulation; selection of preferred policy option; policy implementation and evaluation*)
3. What type of evidence has been used at the different stages of policy development? (*probe for use of different types of evidence at the different stages*)
4. What were the favorable factors that facilitated uptake of evidence at the different stages?
5. What were the barriers to uptake of evidence at the different stages?
6. Who are the stakeholders in policy development and implementation with respect to malaria drug policy change and implementation?
7. What were their positions in the process malaria drug policy change and implementation? (probe for whether strong or weak in terms of influence and whether supportive or opposed, networking)
8. Is there any final comment you would like to make regarding improving uptake of evidence in policy development?

Thank you for this interview.

**In case of any further questions or clarifications; please contact:
Juliet Nabyonga Orem**

**E: mail:** [**julienabyonga@yahoo.com**](mailto:julienabyonga@yahoo.com)

**Phone no. 0772 488 596**

**In-depth interview guide for service providers:**

The health sector in Uganda is underfunded and we need to use available funding optimally. This can only be achieved if we base our policy decisions and implementation on available evidence and ensure that we put money where returns and maximized. I would like to get your views on whether evidence has/has not been used and reasons why, level of importance, barriers and facilitating factors to uptake of evidence at the different stages of policy development. ***My focus is on malaria drug policy change and implementation.***

I have defined research broadly to include, research studies (both published and unpublished) and monitoring and evaluation studies, undertaken within the country by research institutions, universities, donors, civil society and government statistical units and; Ministry of Health reports.

You have been selected as one of the respondents because of your involvement in policy implementation in the areas of interest is the malaria drug policy change and implementation.

May I use the recorder to record your responses?

Name of the interviewer: ________________________________________________

Date and time of the interview: ____________________________________________

*Details of the respondent:*

Name of respondent: _________________________________________________________

Title of respondent/Designation:__________________________________________

Stakeholder group/subsector respondent belongs to: ________________________________

Could you please briefly describe to me your current role and work responsibilities in this organization/institution *(probe for qualifications, research/policy involvement, and work experience*

Duration in that position: ________years; ___________months;______________

1. What do you consider as evidence suitable for informing policy development and implementation? (*probe for type of evidence, local vs international evidence, routine monitoring and evaluation, operational research, systematic reviews, social science, biomedical research*)
2. Has evidence been used in policy implementation with respect to malaria drug policy change and implementation? (*probe for use of evidence at the different stages; agenda setting; policy formulation; selection of preferred policy option; policy implementation and evaluation*)
3. What type of evidence has been used in policy implementation? (*probe for use of different types of evidence at the different stages*)
4. What were the favorable factors that facilitated uptake of evidence?
5. What were the barriers to uptake of evidence?
6. Who are the stakeholders in policy implementation with respect to malaria drug policy change and implementation?
7. What were their positions in the process of malaria drug policy change and implementation? (probe for whether strong or weak in terms of influence and whether supportive or opposed, networking)
8. Is there any final comment you would like to make regarding improving uptake of evidence in policy development?

Thank you for this interview.

**In case of any further questions or clarifications; please contact:
Juliet Nabyonga Orem**

**E: mail:** [**julienabyonga@yahoo.com**](mailto:julienabyonga@yahoo.com)

**Phone no. 0772 488 596**
