# Supplementary material for: Malaria treatment policy change in Uganda: what role did evidence play?
Source: Malar J. 2014 Sep 2;13:345. doi: 10.1186/1475-2875-13-345 (PMC4164770; doi:10.1186/1475-2875-13-345)
Supplement: Supplementary file 2 — Additional file 2: Guideline for document review. Guide used in reviewing documents. (DOCX 12 KB) [file 12936_2014_3385_MOESM2_ESM.docx]

# Guideline for document review

Documents to be reviewed: policies, implementation guidelines, minutes of meetings, government position papers, strategic and annual plans, budget framework papers and identified evidence available over the timeline of policy development. The timeline of the policy will determine the period over which documents will be reviewed.

Regarding evidence, this will be defined broadly to include, research studies (both published and unpublished) and monitoring and evaluation studies, undertaken within the country by research institutions, universities, donors, civil society and government statistical units and; Ministry of Health Reports.

***Documents reviewed***

- When was the document developed?
- Has evidence been referenced in the document?
- Was evidence discussed? (In case of minutes of meetings)
- What type of evidence? (Research, routine monitoring, systematic review, local/international evidence,
- What is the source of the referenced evidence?
- How many times has evidence been cited? What type of evidence? What is the source of evidence?
- Is there contradiction/consistence between available evidence and agreed policy/implementation decision?
- What do the show with regards, to the MRT, institutional strengthening, research characteristics and partnerships?

***Review of available evidence (research reports, monitoring and evaluation reports)***

- When was the research finalized?
- What were the key findings?
- What were the recommendations?
- Who commissioned the research?
