# Supplementary material for: Malaria treatment policy change in Uganda: what role did evidence play?
Source: Malar J. 2014 Sep 2;13:345. doi: 10.1186/1475-2875-13-345 (PMC4164770; doi:10.1186/1475-2875-13-345)
Supplement: Supplementary file 3 — Additional file 3: Documents reviewed. Details of milestones and list of documents reviewed. (DOCX 24 KB) [file 12936_2014_3385_MOESM3_ESM.docx]

# Documents reviewed

| **Mile stone** | **Year document was developed** | **Documents reviewed** |
| --- | --- | --- |
| 1. Initiation of discussion on need for policy change from CQ/SP to ACT; January 2004 |  |  |
| 1. Global Fund round 4 grant proposal on procurement of ACTs for malaria treatment submitted; April 2004 |  |  |
| 1. Consensus meeting agrees on AL as first line ACT for malaria treatment and AQ/AS as alternate first line; May 2004 | March 2004 | Proposal for review of the Anti malarial drug policy for Uganda |
|  | May 2004 | Summary report of the case management working group meeting to review the antimalaria drug policy for Uganda |
|  | May 2004 | Report of the malaria case management working group meeting to review the malaria treatment policy for Uganda |
|  | May 2004 | Concept paper for implementing ACT policy |
| 1. Consensus meeting chaired by the Director General agrees on use of ACTs at community level as well; June 2004 |  |  |
| 1. Ministry of health top management approval of the drug policy change; June 2004 |  |  |
| 1. Launch of task force and its sub-committees; August 2004 | August 2004 | Report of the supply chain management taskforce |
|  | August 2004 | Report of M & E task force |
|  | August2004 | Treatment and training working group: Treatment guidelines and training approaches |
|  | August 2004 | IEC working group: Communication strategy for treatment of uncomplicated malaria using AL |
|  | January 2005 | Communication to WHO by MoH (DGHS) requesting for ordering of ACTs |
| 1. Signing of the Global fund round 4 grant; April 2005 |  |  |
| 1. Registration of Coartem by National Drug Authority; June 2005 |  |  |
|  | July 2005 | Minister of Health meeting with Ambassadors on malaria drug procurement using GFATM funds (Wrote by DFID health adviser) |
| 1. Suspension of Global fund grants to Uganda; August, 2005 | August 2005 | Report on the review to update the draft malaria treatment guidelines |
|  | 2005 | National policy on malaria treatment |
|  | 2005 | Management of uncomplicated malaria; a practical guide for all health workers, 3rd edition |
| 1. Global fund suspension lifted November 2005 |  |  |
|  | December 2005 | Implementation manual for the national policy on malaria treatment, second edition |
| 1. 1^st^ shipment of AL received in the country, January 2006 |  |  |
|  | 2006 | Implementation guidelines for the HBMF strategy 2nd Edition |
|  | March 2006 | WHO press release - WHO calls for immediate halt to provision of single-dose artemisinin malaria pills. New malaria treatment guidelines issued by WHO |
| 1. Official launch of new treatment policy using ACTs for malaria treatment; April 2006 |  |  |
|  | June 2006 | Documentation of malaria treatment policy change process |
|  | October 2006 | Report of the workshop on strategies for implementation of new antimalarial drug policy |

# Research reports:

| **Research reports reviewed** | Commissioning entity | Year | Time after the policy change |
| --- | --- | --- | --- |
| 1. Dr. Frederick K. Kato, October 2006; The malaria treatment policy change process in Uganda | MoH | 2006 | 6 months later |
| 1. 2007, Assessing the availability of the first and second line antimalarials in selected health facilities in Uganda; March | MoH | 2007 | 8 months later |
| 1. MoH, NMCP, Mar. 2007; Analysis of malaria attendances according to AL dosage age groups | MoH | 2007 | 11 months later |
| 1. Zurovac D, Tibendarana J, Nankabirwa J, Ssekitooleko J, Talisuna A, Rwakimari JB; 2007; Evaluation of outpatient malaria case-management under artemether-lumefantrine treatment policy in Uganda; | Partnership | Aug 2007 | 1 Year later |
