# Supplementary material for: Malaria treatment policy change in Uganda: what role did evidence play?
Source: Malar J. 2014 Sep 2;13:345. doi: 10.1186/1475-2875-13-345 (PMC4164770; doi:10.1186/1475-2875-13-345)
Supplement: Supplementary file 4 — Additional file 4: Factors that facilitated the uptake of evidence in the malaria treatment policy change. Details of facilitatory factors under the different themes. (DOCX 20 KB) [file 12936_2014_3385_MOESM4_ESM.docx]

# Factors that facilitated uptake of evidence in the malaria treatment policy change

|  |  |  | **Public sector** | | | | **Private sector** | | |  |  |
| --- | --- | --- | --- | --- | --- | --- | --- | --- | --- | --- | --- |
|  |  | **Donors** | **MoH** | **NMS** | **NDA** | **Service providers** | **CSO** | **Pharmaceutical company** | **Media** | **Researchers** | **Total** |
| **Characteristics**  **of available evidence** | Availability of local evidence of high quality | 3 | 6 | 1 |  | 7 | 1 | 1 |  | 1 | **20** |
|  | Availability of in-country competent researchers | 1 | 4 |  |  | 2 | 1 | 1 |  | 1 | **10** |
|  | Consistent results from multiple study undertaken by different researchers | 1 | 3 |  |  | 2 |  | 1 |  |  | **7** |
|  | Evidence generated by credible international researchers/Regional networks | 1 | 2 |  |  | 1 | 1 |  |  |  | **5** |
|  | Consensus on research results | 1 | 3 |  |  |  |  |  |  | 1 | **5** |
|  | Dissemination of evidence |  |  |  |  | 2 | 1 | 1 |  |  | **4** |
|  | Evidence was readily available | 2 |  | 1 |  |  |  |  |  |  | **3** |
|  | community cries | 1 | 2 |  |  |  |  |  |  |  | **3** |
|  | **Total number of responses** | **10** | **20** | **2** |  | **14** | **4** | **4** |  | **3** | **57** |
|  |  |  |  |  |  |  |  |  |  |  |  |
| **MoH institutional capacity to lead the KT process** | Leadership and willingness of MoH to use evidence | 1 | 5 |  | 1 | 1 |  |  |  | 2 | **10** |
|  | MoH involvement in research studies |  | 1 |  |  | 1 | 1 |  |  | 1 | **4** |
|  | Culture of using evidence in changing treatment policies by the MoH |  | 2 |  |  |  |  |  |  | 1 | **3** |
|  | Political will to invest in health |  |  |  | 1 |  | 2 |  |  |  | **3** |
|  | **Total number of responses** | **1** | **8** |  | **2** | **2** | **3** |  |  | **4** | **20** |
|  |  |  |  |  |  |  |  |  |  |  |  |
|  | Availability of platforms for discussing evidence and policy development | 3 | 3 |  | 1 | 2 | 1 | 1 |  | 1 | **10** |
|  | Availability of structures within MoH |  | 2 |  |  | 1 |  |  |  |  | **3** |
| **Partnerships for KT** | Interest of stakeholders to see uptake of the policy |  | 2 |  |  |  |  |  |  |  | **2** |
|  | Civil society involvement |  |  |  |  |  | 1 |  |  |  | **1** |
|  | **Total number of responses** | **3** | **7** |  | **1** | **3** | **2** | **1** |  | **1** | **18** |
|  |  |  |  |  |  |  |  |  |  |  |  |
|  | Training of health workers on implementation of the new policy | 1 | 2 |  |  | 4 |  |  |  |  | **7** |
| **Availability of tools and inputs to implement evidence** | Availability of funding from the Global fund to fund the new policy | 1 | 2 |  |  | 1 | 1 |  |  |  | **5** |
|  | Guidelines were available |  |  |  |  | 4 |  |  |  |  | **4** |
|  | Availability of inputs and tools to implement the policy |  | 1 |  |  |  |  |  |  |  | **1** |
|  | **Total number of responses** | **2** | **5** |  |  | **9** | **1** |  |  |  | **17** |
| **WHO** | WHO Intervention | 2 | 2 |  |  |  | 1 |  |  | 1 | **6** |
|  | WHO's influence, being in support of the evidence | 1 | 2 |  |  |  |  |  |  |  | **3** |
|  | **Total number of responses** | **3** | **4** |  |  |  | **1** |  |  | **1** | **9** |
